# Awake intubation and extraluminal use of Uniblocker for one-lung ventilation in a patient with a large mediastinal mass a case report

**DOI:** 10.1186/s12871-020-01041-7

**Published:** 2020-05-25

**Authors:** Zhuo Liu, Qianqian Jia, Xiaochun Yang

**Affiliations:** Department of Anesthesiology, The First Hospital of Qinhuangdao, N.O. 258, Wenhua Road, Qinhuangdao, Hebei China

**Keywords:** One-lung ventilation, Awake intubation, Extraluminal use of Uniblocker, Mediastinal mass

## Abstract

**Background:**

The anesthesia of patients with large mediastinal mass is at high-risk. Avoidance of general anesthesia in these patients is the safest option, if this is unavoidable, maintenance of spontaneous ventilation is the next safest technique. In these types of patients, it is not applicable to use double-lumen tube (DLT) to achieve one-lung ventilation (OLV) because the DLT has a larger diameter and is more rigid than single-lumen tube (SLT), so the mass may rupture and bleed during intubation. Even using a bronchial blocker, a small size of SLT is required for once the trachea collapses the SLT can pass through the narrowest part of trachea. However, it is difficult to control the fiberoptic bronchoscopy (FOB) and the bronchial blocker simultaneously within the lumen of a small size SLT with traditional intubation methods.

**Case presentation:**

The current study presented a 66 years old female patient with a large mediastinal mass that presented with difficulty breathing when lying flat. In this case, we combined use of dexmedetomidine and remifentanil to preserve the patient’s spontaneous ventilation during intubation and achieved one-lung ventilation with extraluminal use of Uniblocker.

**Conclusions:**

Extraluminal use of Uniblocker and maintenance of spontaneous ventilation during intubation may be an alternative to traditional methods of lung isolation in such patients with a large mediastinal mass.

## Background

Large mediastinal masses can cause airway collapse and hemodynamic collapse and these feared complications occur particularly during positional changes and with induction of anesthesia or muscle relaxation, which is why the anesthesia of these patients with large mediastinal mass is at high-risk [1]. We presented a single case report of a patient whose airway management was especially challenging.

## Case presentation

**Fig. 1:**
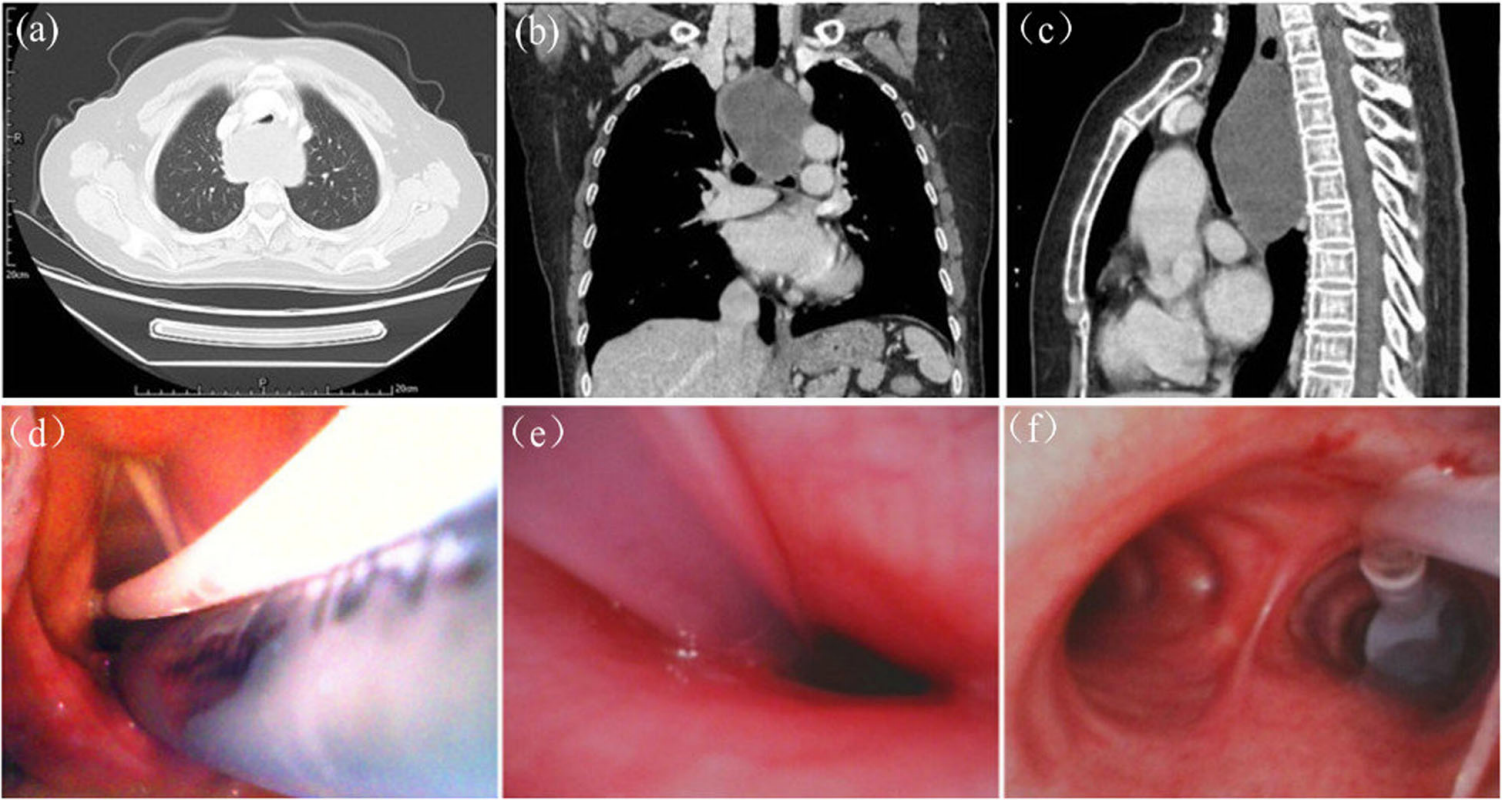
**a** The image of the mass in the transverse position; **b** The image of the mass in the coronary position; **c** The image of the mass in the sagittal position; **d** The Uniblocker and single lumen tube passed the glottis; **e** The Uniblocker passed through the narrowest part of the trachea; **f** the cuff of the Uniblocker located below the carina

A 66 years old female patient, weight 52 kg, height 150 cm was scheduled for mediastinal mass resection surgery. Because the mediastinal mass had been compressed the weakened trachea and interfered with the patient’s breathing, so the surgery needed to be performed as soon as possible. The patient had a general anaesthetic 14 years ago for laparoscopic cholecystectomy without complications. Pre-operative blood pressure (BP) was 101/72 mmHg, heart rate (HR) was 85 min^− 1^, respiratory rate (RR) was 20 per minute and SpO_2_ was 94%. Preoperative chest computed tomographic (CT) scans showed that a large mediastinal mass (10.1 cm × 7.4 cm × 4.9 cm) compressed the trachea and carina. The narrowest part of the trachea was located at 4.9 cm above the carina, where the cross section of the trachea was a fissure (0.45 cm × 1.41 cm) (Fig. 1a,b,c).

The patient without premedication and received standard monitoring in the operating room. After preoxygenation the patient was intravenously injected with midazolam 0.03 mg·kg^− 1^ and then an arterial catheter and an internal jugular vein catheter were placed under local anesthesia. A transtracheal injection of 1% lidocaine (3-4 ml) was administered and the patient was suggested to open mouth then the oral cavity and hypopharynx mucosa were sprayed with 1% lidocaine.

After intratracheal surface anesthesia, the patient was received dexmedetomidine at a loading dose of 1 μg·kg^− 1^ (the infusion was completed in 10 min) then remifentanil at a loading dose of 0.5 μg·kg^− 1^, followed by a continuous infusion at a speed of 0.1 μg·kg^− 1^·min^− 1^. During this process, the patient was received continuous oxygen by mask. After deep sedation (patient breathing spontaneously but cannot be awakened by calling her name), the intubation was performed and the steps were as follows: First, inserted a Uniblocker (9-French) into the trachea via a visual laryngoscope and advanced the Uniblocker toward the right main-stem bronchus after the tip passed the glottis; Second, inserted a single lumen tube (SLT, inner-diameter 6.0 mm) into the trachea until the cuff of SLT passed the glottis (Fig. 1d,e); Third, fixed the Uniblocker and SLT to the patient’s mouth separately with a cloth tape; Finally, inserted the fiberoptic bronchoscopy (FOB, external diameter 3.8 mm, MDHAO Medical Technology, Zhuhai, China) into the lumen of SLT to adjust the Uniblocker to optimal position.

After 4 attempts of adjustment, the Uniblocker to optimum position (Fig. 1f). Anesthesia maintenance with 1–2% sevoflurane and continuous infusion of remifentanil and propofol at a speed of 0.1–0.2 μg·kg^− 1^·min^− 1^ and 30-80 μg·kg^− 1^·min^− 1^. The narrowest part of the trachea was monitored: if there was a sudden increase of peak airway pressure, the FOB would be inserted into the tube to detect the stenosis of trachea; if the airway collapsed and the SLT could pass through the narrowest part of the trachea via FOB then the SLT would be advanced through the stenosis as soon as posible; If the airway collapsed after anesthesia and the SLT could not be advanced through the narrowest part of the trachea, our plan is to change the patient’s position and use high frequency jet ventilation via the Uniblocker to maintain the patient’s oxygen supply then the emergent extracorporeal circulation would be established and the operation would be performed under extracorporeal circulation; If the airway collapsed intraoperative we would recommend the surgeon to lift up the mass or drain the cyst fluid as soon as possible then advance the SLT through the narrowest part of trachea.

During the operation, the airway was not collapsed. After 1.5 h, the mass was successfully removed without any complications and the SLT was also successfully removed in the post anesthesia care unit.

## Discussion and conclusions

The most feared complications of mediastinal mass resection surgery are airway collapse and hemodynamic collapse. Avoidance of general anesthesia is a prevailing recommendations in such patients [2–5]. If general anesthesia is required, avoidance of paralytic agents and maintenance of spontaneous ventilation are emphasized [2–5]. In this case, we combined use of dexmedetomidine and remifentanil to preserve the patient’s spontaneous ventilation during intubation.

Large mediastinal masses increase the complexity of one lung ventilation. In this patient, the chest CT revealed that the trachea was severely compressed and the narrowest part of the trachea was only 0.45 cm, so the DLT may not pass through the narrowest part of the trachea (Fig. 1e) and bronchial blockers (BBs) may be more suitable for this patient [6]. However, even using BBs, a small size of SLT should be chosen for this patient, for once the airway was obstructed, the SLT could be advanced through the narrowest part of trachea via FOB. With the conventional intubation method, both the BBs and FOB are inserted into the lumen of the SLT then the BBs are guided to the optimal position, so it is difficult to contral the FOB and rotate BBs simultaneously in the lumen of a small size SLT. Compared with conventional intubation method, extraluminal use of BBs has more advantageous, especially in this case: First, with this method, we were able to choose a small size of SLT (ID 6.0 mm), so the SLT might be easy to pass through the narrowest part of trachea via FOB once the airway collapsed. Second, the Uniblocker could be easily positioned without the interference of FOB and the limitations of narrow spaces of SLT when adjusted the Uniblocker to the optimal position, especially in this case the trachea was compressed and displaced. Third, the lumen of SLT was unobstructed, so a suction catheter could be easily inserted into the SLT to clear the hemorrhage once the mass ruptures and bleeds.

In conclusion, this case highlights that in the patient with large mediastinal masses, extraluminal use of Uniblocker and the combination use of dexmedetomidine and remifentanil to preserve the patient’s spontaneous ventilation during intubation increase the patient’s safety and this novel method may be an alternative to traditional methods of lung isolation in the patients with airway stenosis.

## Data Availability

The datasets are available from the corresponding author on request.

## Abbreviations

OLV: One-lung ventilation
DLT: Double-lumen tube
SLT: Single-lumen tube
CT: Computed tomography
FOB: Fiberoptic bronchoscopy
